# *Opisthorchis felineus* infection provokes time-dependent accumulation of oxidative hepatobiliary lesions in the injured hamster liver

**DOI:** 10.1371/journal.pone.0216757

**Published:** 2019-05-14

**Authors:** Mariya Y. Pakharukova, Oxana G. Zaparina, Yaroslav K. Kapushchak, Nina V. Baginskaya, Viatcheslav A. Mordvinov

**Affiliations:** 1 Laboratory of Molecular Mechanisms of Pathological Processes, Institute of Cytology and Genetics, Siberian Branch of Russian Academy of Sciences, Novosibirsk, Russia; 2 Department of Natural Sciences, Novosibirsk State University, Novosibirsk, Russia; 3 Novosibirsk State Medical University, Novosibirsk, Russian Federation, Novosibirsk, Russia; North-Eastern Hill University, INDIA

## Abstract

Opisthorchiasis caused by food-borne trematode *Opisthorchis felineus* is a substantial public health problem, with 17 million persons infected worldwide. This chronic disease is associated with hepatobiliary inflammation, cholangiocyte dysplasia, cholangiofibrosis, intraepithelial neoplasia, and even cholangiocarcinoma among chronically infected individuals. To provide first insights into the mechanism by which *O*. *felineus* infection causes precancerous liver lesions, we investigated the level of oxidative stress (lipid peroxidation byproducts and 8-hydroxy-2′-deoxyguanosine) as well as the time course profiles of chronic inflammation and fibrogenesis markers in the dynamics of opisthorchiasis from 1 month to 1.5 years postinfection in an experimental model based on golden hamsters *Mesocricetus auratus*. For the first time, we showed that *O*. *felineus* infection provokes time-dependent accumulation of oxidative hepatobiliary lesions in the injured liver of hamsters. In particular, over the course of infection, lipid peroxidation byproducts 4-hydroxynonenal and malondialdehyde were upregulated; these changes in general correlate with the dynamics of hepatic histopathological changes. We detected macrophages with various immunophenotypes and elevated levels of CD68, COX2, and CD163 in the *O*. *felineus*–infected animals. Meanwhile, there was direct time-dependent elevation of TNF-α (R = 0.79; p < 0.001) and CD163 protein levels (R = 0.58; p = 0.022). We also provide quantitative data about epithelial hyperplasia marker CK7 and a marker of myofibroblast activation (α smooth muscle actin). Our present data provide first insights into the histopathological mechanism by which *O*. *felineus* infection causes liver injuries. These findings support the inclusion of *O*. *felineus* in Group 1 of biological carcinogens.

## Introduction

More than 20% of cancer cases in the developing world are caused by infections. The International Agency for Research on Cancer (IARC) recognizes the infection with fish-borne trematodes as Group 1 biological carcinogens including infection with liver flukes *Opisthorchis viverrini* and *Clonorchis sinensis*, which are considered definitive causes of cholangiocarcinoma in endemic regions [1, 2].

*O*. *felineus* is a fish-borne trematode and is one of the causative agents of opisthorchiasis [3, 4]. In the past, *O*. *felineus* occurred primarily within the territory of the former USSR. Increasingly, it has been found in other European regions like Italy [5, 6]. It is estimated that worldwide, 1.6 million out of 17 million patients infected with Opisthorchiidae trematodes have opisthorchiasis resulting from infection with *O*. *felineus* [4, 5]. The chronic disease is associated with hepatobiliary inflammation, cholangiocyte dysplasia, cholangiofibrosis, intraepithelial neoplasia, and presumably cholangiocarcinoma among chronically infected individuals [4]. Direct evidence supporting a role of *O*. *felineus* infection as a risk factor of cholangiocarcinoma is still scarce [2, 4], and this topic is not well studied [4].

Our previous papers have presented findings about infection of hamsters, and these data support the inclusion of *O*. *felineus* in the list of Group 1 carcinogens and indicate that this pathogen is a definitive cause of cholangiocarcinoma [7, 8]. In a hamster model of experimental infection with *O*. *felineus*, there is documented evidence of precancerous lesions, e.g., inflammation, fibrosis, and biliary intraepithelial neoplasia of grades BilIN-I, -II, and -III. The consonance of these histopathological changes has revealed that *O*. *felineus* infection in this rodent model induces precancerous lesions conducive to malignancy. Additionally, oxysterol-like metabolites in the *O*. *felineus* worms, and in the bile, serum, and urine of the infected hamsters were detected by liquid chromatography with mass spectroscopy [8]. Related DNA adducts suggest that infection-associated oxysterols induce chromosomal lesions in host cells. We hypothesized that these helminths produce and excrete metabolites including oxysterols that promote oxidation of host DNA and induce DNA lesions and mutations. These data indicate a crucial role of oxidative stress in the formation of precancerous lesions. Nevertheless, direct evidence of the presence of oxidative stress in the liver tissue during *O*. *felineus* infection and the presence of oxidative DNA damage has not yet been reported.

In this work, we aimed to investigate the level of oxidative stress in the dynamics of opisthorchiasis from 1 month to 1.5 years postinfection by detection of lipid peroxidation (LPO) byproducts in the liver, of the level of oxidative DNA damage by quantitation of 8-hydroxy-2′-deoxyguanosine (8-OHdG) as a predominant form of free-radical–induced oxidative DNA lesions, and time-dependent levels of protein markers of chronic inflammation and fibrogenesis in a golden-hamster *(Mesocricetus auratus)* model of opisthorchiasis.

## Materials and methods

### Ethics statement

Territories where fishing took place were neither conservation areas nor private, nor otherwise protected; hence, no fishing permits were required. The fish species collected are not considered endangered or rare, and the fishing methods complied with the Federal Law N166-F3 of 20.12.2004 (ed. 18.07.2011), “Fishing and conservation of water bio-resources”.

All the procedures were in compliance with EU Directive 2010/63/EU for animal experiments. Study design protocols and standard operating procedures (the hamsters and the fish) were approved by the Committee on the Ethics of Animal Experiments of the Institute of Cytology and Genetics of the Siberian Branch of the Russian Academy of Sciences (ICG SB RAS; Permit Number: 42 of 25.05.2018).

### Animal experiments

Golden hamsters (*M*. *auratus*) were purchased from the Specific Pathogen Free (SPF) Animal Facility of the ICG SB RAS. Naturally infected freshwater fish (*Leuciscus idus*) was net-caught in the Ob River near Novosibirsk (Western Siberia) by the research assistant Viktor Antonov (ICG SB RAS) without the use of chemicals. Euthanasia of fish was perform by decapitation. *O*. *felineus* metacercariae were extracted as described previously [9]. Briefly, the fish tissue was immersed in a digestion solution (0.9% NaCl in distilled water with 1% pepsin and 1% HCl, pH 2.0) for 2 h at 37°C followed by filtration. After several washes with normal saline, the metacercariae were collected and identified under a light microscope.

To assess the progression of opisthorchiasis-associated pathological changes, thirty-seven 2-month-old male *M*. *auratus* hamsters were randomly subdivided into two groups: uninfected and infected orally with 75 metacercariae of *O*. *felineus*. The hamsters were housed at one per cage under conventional conditions and were provided with standard rodent feed (PK-120-1; Laboratorsnab, Ltd., Moscow, Russia) and water *ad libitum*. All hamsters were observed daily for signs of illness, injury, or abnormal behavior by a person trained to recognize such signs. Food and water availability, the macroenvironment (temperature, humidity, noise, light intensity, cleanliness) was also evaluated daily. No unexpected deaths of animals were registered during this study.

Four hamsters from the uninfected group and 4–5 hamsters from the infected group were euthanized and necropsied at 1, 3, 6, 11, and 18 months postinfection (p.i.). Euthanasia of hamsters was performed by carbon dioxide inhalation, and every effort was made to minimize the suffering of the hamsters.

### Sample collection and histopathological analysis

Hamsters were euthanized using carbon dioxide, after which blood was collected by cardiac puncture. Blood was centrifuged at 3000 × *g* for 20 min at 4°C to obtain serum. Their urine was collected by bladder puncture. The serum and urine samples were aliquoted and stored at −80°C. For an immunohistochemical assay, the livers were immediately fixed in 4% paraformaldehyde in phosphate-buffered saline (PBS). The fixed livers were sliced at 7 or 10 μm (n = 4 to 5; 4–6 serial tissue sections per animal) on a Microm HM-505 N cryostat.

For histological analysis, the liver was carefully dissected and placed in 10% buffered formalin (Biovitrum, Russia). After fixation for 7 days at 4°C, the specimens were dehydrated in a graded series of ethanol solutions and then absolute ethanol, cleared in xylene, and soaked in melted paraffin. After that, we embedded the specimens in paraffin using Microm (Microm, UK). Four-μm-thick slices were prepared by means of a microtome. The tissue slices were stained with hematoxylin and eosin or Van Gieson’s dye by the standard methods and were examined under a light microscope (Axioskop 2 Plus; Zeiss, Germany). Pathological changes were assessed in two lobes of the liver of each animal.

### Immunohistochemistry

Immunohistochemical analysis was performed as described elsewhere [10]. Antibodies and dilutions used in this study include anti–4-hydroxynonenal (1:150 dilution; cat. # ab48506, Abcam), anti-malondialdehyde (1:300; cat. # ab6463, Abcam), anti-CD68 (1:50; cat. # ab201340; # ab53444, Abcam), and anti-COX2 (1:50; cat. # ab201340, Abcam and 1:50; # sc-1747R, Santa Cruz, USA), antibodies; with the respective secondary antibodies: a cyanine 3–conjugated goat anti-mouse IgG (H+L) cross-adsorbed antibody, (1:500; cat. #M30010 Invitrogen, USA) or a GFP-conjugated AffiniPure goat anti-rabbit IgG (H+L) antibody (1:1000; cat. #111-095-003, Jackson AB, USA) and mouse anti-rabbit IgG-CFL 555: sc-516249, Santa Cruz, USA). The slices were coverslipped with the Fluoro-shield mounting medium containing 4′,6-diamidino-2-phenylindole (DAPI; cat. # F6057, Sigma-Aldrich, USA) and examined under an Axioplan 2 microscope (Zeiss, Germany).

### Western blotting

Immunoblotting was performed as described elsewhere [11]. Antibodies and dilutions employed in this study include anti–TNF-α (1:2000; cat. # 3114560, Sony), anti–β-actin (1:2000; cat. # ab8226, Abcam), anti–α smooth muscle actin (α-SMA; 1:2000; cat. # ab7817, Abcam), anti–cytokeratin 7 (1:2000; cat. # ab9021, Abcam), and anti-CD68 (1:2000; cat. # ab201340, Abcam) antibodies. Quantitative densitometric analyses were performed on digitized images of immunoblots in the Quantity One software (Bio-Rad, USA).

### 8-hydroxy-2'-deoxyguanosine level

Urinary 8-hydroxy-2' -deoxyguanosine (8-OHdG) level was measured using 8-OHdG ELISA kit (cat. # ab201734, Abcam).

### Statistical analysis

The data were subjected to statistical analysis in the Statistica 6.0 software (Statsoft, USA). The Shapiro-Wilk test was used to check whether the distribution follows the normal pattern. One-way ANOVA F-test with the Newman–Keuls *post hoc* analysis was applied to significant main effects and interactions to assess the differences between some sets of means. The time–effect relation was assessed by linear regression analysis.

## Results

### Histological findings

**Fig 1:**
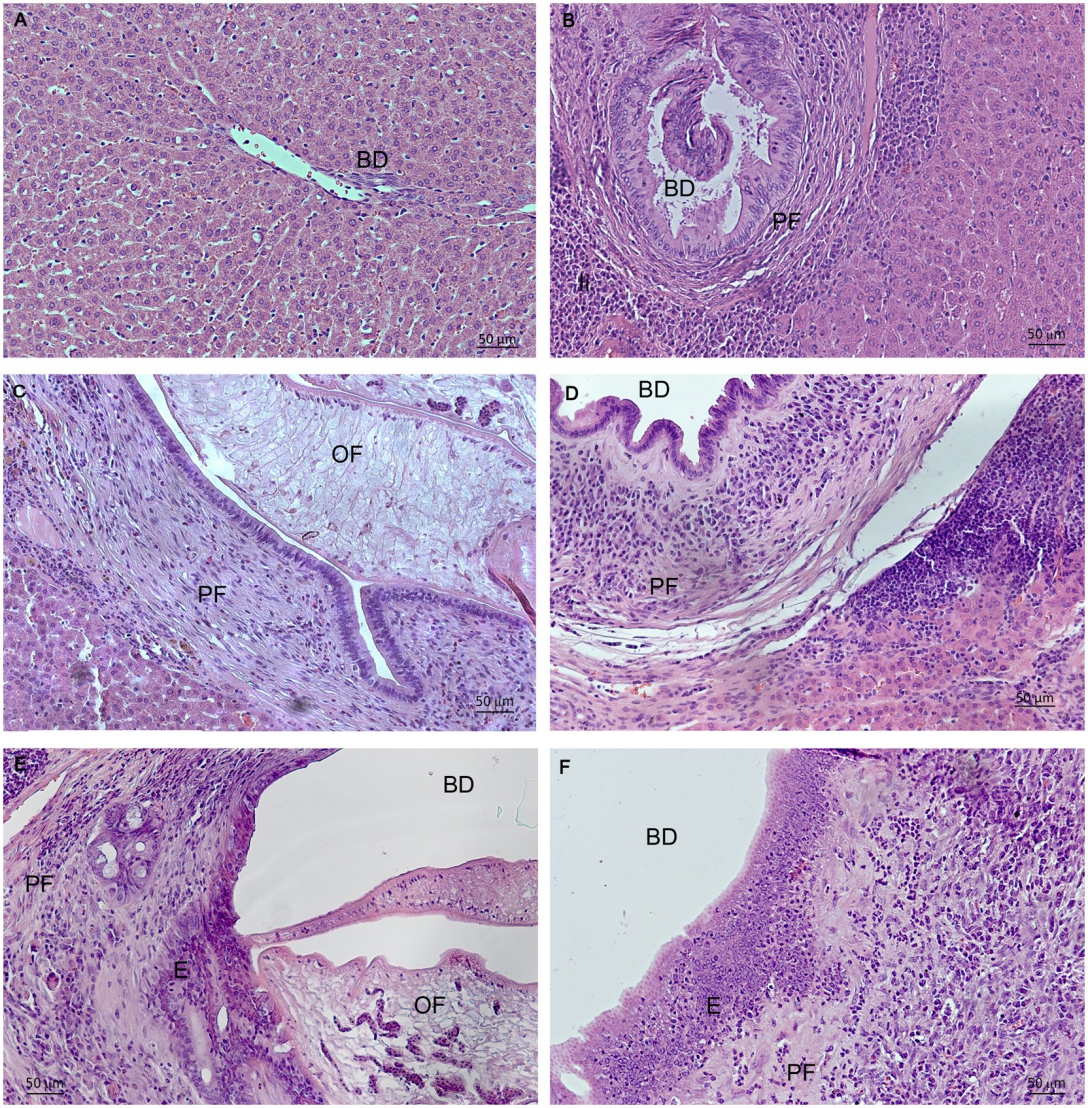
Biliary histological features observed in the liver biopsies from control and infected hamsters (hematoxylin and eosin staining). **A. Uninfected. B. One month p.i. C. Three months p.i. D. Six months p.i. E. Eleven months p.i. F. 1.5 years p.i**.; BD: bile ducts, PF: periductal fibrosis; OF: *Opisthorchis felineus*; E: epithelium of bile ducts; Ii: inflammatory infiltration.

Histological examination of the livers of infected hamsters revealed that the bile ducts were lined by an epithelium with enlarged nuclei, loss of polarity, and low-grade dysplasia (Fig 1). Among the epithelial cells in the bile ducts, mucin-secreting cells were visible well. A transition from the normal duct epithelium to an atypical lesion was suggestive of cancerization of the duct or ductile (Fig 1). Granulomatous inflammation and mononuclear-cell and eosinophil infiltration of the portal area in the periductal tissue and liver parenchyma were also evident (Fig 1). The major histological changes observed in the gallbladder and extrahepatic bile ducts included adenomatous hyperplasia, epithelial hyperplasia, and chronic inflammation. In the course of the experiment, enhancement of bile duct cell proliferation and thickening of the periductal fibrosis in the hyperplastic bile ducts were observed. Severe inflammation started at 1 month p.i. and persisted throughout the p.i. time in our study. In general, it was noted that the severity of the infection and the magnitude of pathological changes gradually increased from the start of the infection to 1.5 years p.i. Cholangiofibrosis was found in animals starting from 11 months p.i. Although no gross appearance of a tumor mass of CCA was demonstrated, histopathological features of CCA were detected in two of five hamsters in the 11th month p.i.

### Oxidative lesions

The detrimental effects of LPO can be attributed to cytotoxic and mutagenic properties of the secondary aldehyde species (MDA and HNE) that result from LPO chain reactions [12].

**Fig 2:**
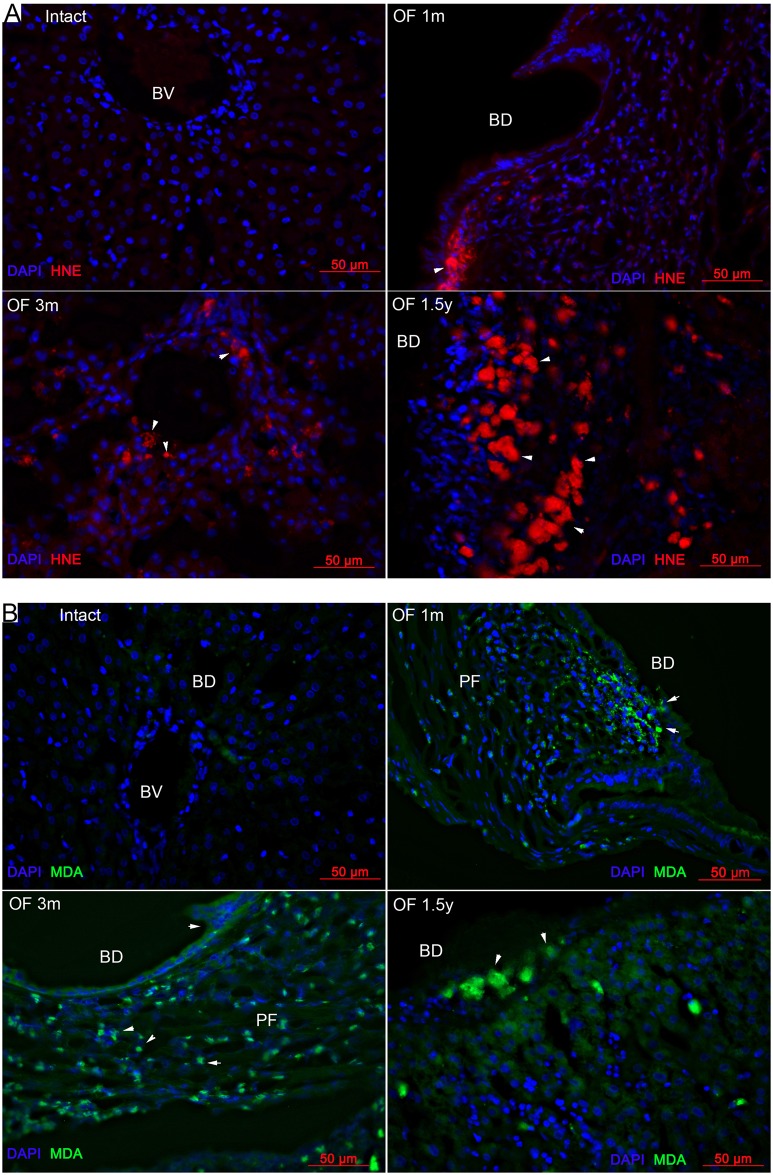
Oxidative-stress–induced lesions in the liver of hamsters with opisthorchiasis (uninfected and at 1 and 3 months and 1.5 years p.i. with *O*. *felineus)*. **A. 4-Hydroxynonenal (HNE). B. Malone dialdehyde (MDA)**; BD: bile ducts, PF: periductal fibrosis, BV: blood vessel. A specific signal of the antigen is indicated with an arrow.

In the liver of uninfected animals, HNE was hardly detectable. By contrast, in the liver of the hamsters with opisthorchiasis, the signal of HNE was substantially present (Fig 2) already at 1 month p.i. The HNE signal was mainly associated with cells in the periductal area and near blood vessels. The intensity of the HNE signal increased with the duration of the infection. In the liver of hamsters with opisthorchiasis at 1.5 years p.i., the strongest HNE signal was detected. The signal corresponding to HNE at 1.5 years p.i. was visible not only in cells in the periductal area but also in the liver parenchyma and epithelial cells of bile ducts.

MDA content in the liver of infected animals also underwent similar time-dependent changes, in particular, although there was almost no signal in the liver of uninfected animals, starting from 1 month p.i., MDA was detectable in relevant tissues, especially in the periductal area, in liver inflammatory infiltrates. Nevertheless, it was possible to notice the staining of MDA in the epithelial cells of the bile duct, starting from 1 month p.i. After comparison of the signals of these two LPO byproducts at 1 month p.i., it should be noted that large numbers of inflammatory cells immunostained for HNE, whereas only a few cells immunostained for MDA. The MDA signal increased with the duration of animal infection. In hamsters with opisthorchiasis at 1.5 years p.i., the highest intensity of the MDA signal was detected in the liver. At this time, product MDA is already found in the liver parenchyma.

The presence of lipid peroxidation products in the liver of infected animals indicates the highly elevated reactive oxygen species (ROS) production in the liver during opisthorchiasis felinea. Moreover, *O*. *felineus* infection facilitated the accumulation of LPO products in the injured liver in a time-dependent manner.

**Fig 3:**
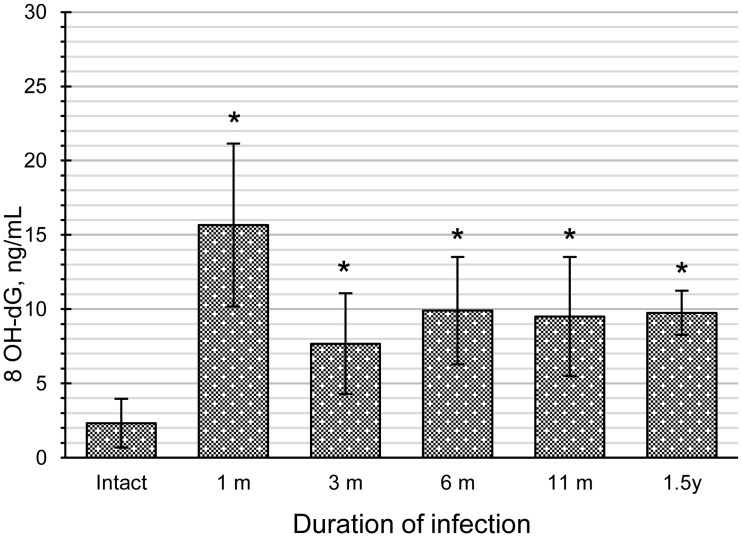
Urinary 8-hydroxy-2′-deoxyguanosine (8-OHdG) levels in hamsters with opisthorchiasis. The data are presented as mean ± SD; *p < 0.05; ***p < 0.001.

It has been reported that increased LPO in intact cells can induce oxidative DNA damage, such as 8-OHdG formation, during the reaction of lipid hydroperoxides with ferric ion [13]. This finding prompted us to test whether the accumulation of LPO byproducts in infected liver tissues would lead to 8-OHdG formation. Indeed, the level of 8-OHdG in urine increased in the animals with opisthorchiasis throughout the experimental infection: from 1 month p.i. (p = 0.0008) to 1.5 years p.i. (p = 0.04; Fig 3).

### Inflammation markers

**Fig 4:**
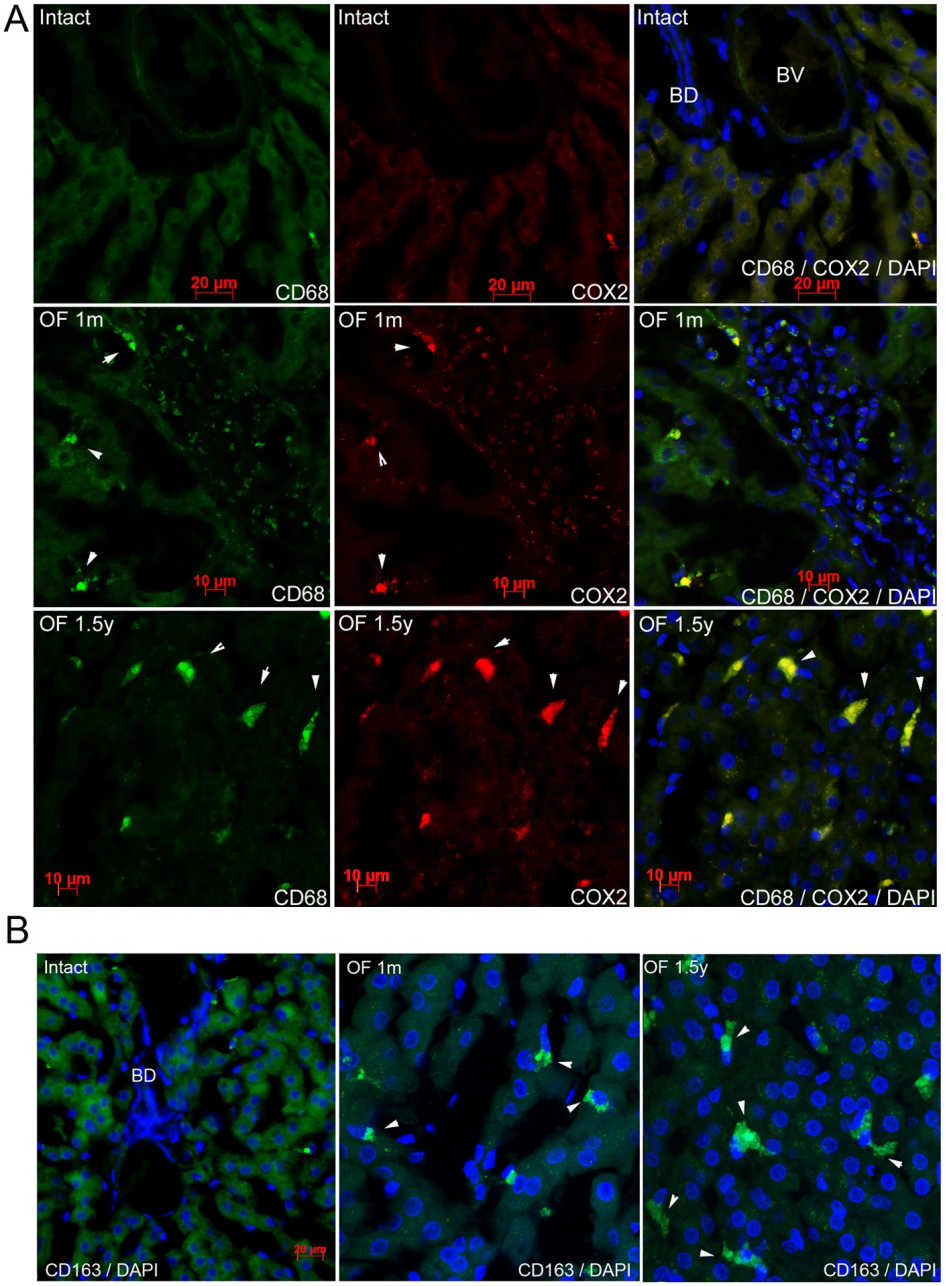
Inflammation markers are upregulated in the liver of infected hamsters. **A. Expression of CD68 and COX2 demonstrated by immunohistochemistry**. A specific signal for each antigen is indicated with an arrow. **B. Expression of CD163 demonstrated by immunohistochemical analysis**. A specific signal for the antigen is indicated with an arrow.

**Fig 5:**
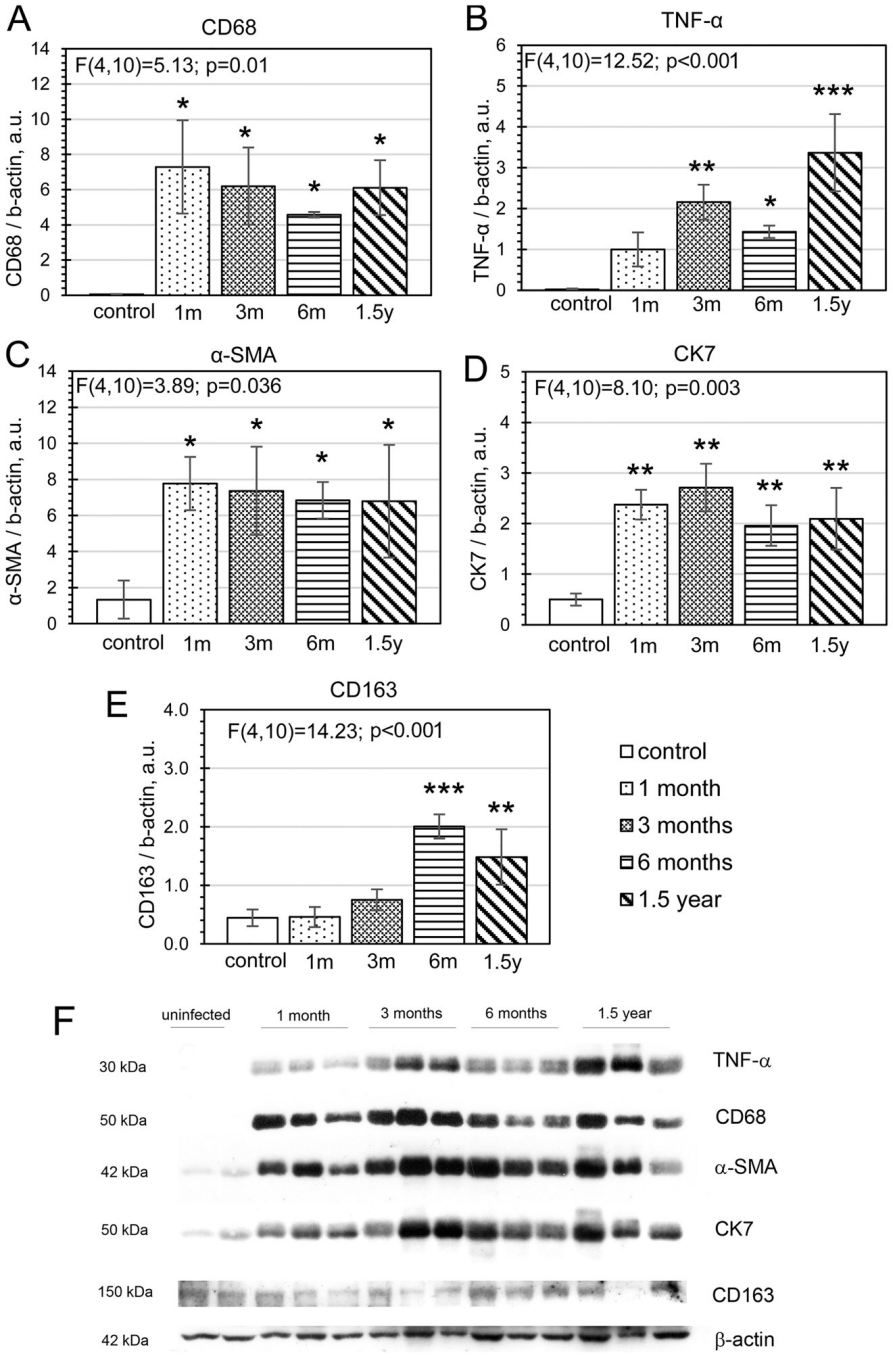
Densitometric analysis of CD68 (A), TNF-α (B), α-SMA (C), CK7 (D), and CD163 (E) protein levels in the liver of uninfected and infected hamsters. **(A–E). F. Western blot analysis of lysates from uninfected and infected animals**. Protein levels were determined by densitometry of immunoblots (**F**), and the results were normalized to β-actin. The data are presented as mean ± SD. *p < 0.05; **p < 0.01; ***p < 0.001. SD is calculated from the results of three independent experiments. CK7: cytokeratin 7; α-SMA: α smooth muscle actin; TNF-α: tumor necrosis factor α; CD68 (cluster of differentiation 68); CD163 (cluster of differentiation 163); SD, standard deviation.

To analyze the relation between oxidative lipid metabolism and to evaluate the inflammatory responses in *O*. *felineus–*infected animals, we tested the levels of proinflammatory and fibrogenic proteins in immunohistochemical (Fig 4A and 4B) and western blotting assays (Fig 5). CD68 (cluster of differentiation 68) is a protein highly expressed by cells of the monocyte lineage and by tissue macrophages. Inducible COX-2 expressed in immune cells is a key player in the initiation of an inflammatory response and in the triggering of production of proinflammatory chemokines and cytokines. COX2 is highly expressed in activated macrophages and participates in immune-response regulation.

In in the liver of uninfected animals, both CD68 and COX2 proteins were hardly noticeable (Fig 4A, upper panel). After only 1 month p.i. (Fig 4A, middle panel), CD68 (green fluorescence) was detected in macrophages. In the same cells, readers can see COX2 (red fluorescence), the signal of which then coincided with two different filters (Fig 4A, right column). Cells expressing both proteins CD68 and COX2 were also found in the liver of animals at 1.5 years p.i. The cells were found not only in the inflammatory infiltrates, but also in the liver parenchyma (Fig 4A, lower panel). These results indicated that the expression of COX2 overlapped with those of LPO byproducts in the infected animals.

The hemoglobin scavenger receptor, CD163 was used to identify the macrophages with alternative activated phenotype [14, 15]. In uninfected animals, the CD163 protein was hardly detectable by immunohistochemistry (Fig 4B, left column), but at 1 month p.i., stand-alone macrophages expressing CD163 were found in the liver. As long as the infection lasted, the number of cells expressing CD163 grew. At 1.5 p.i., the number of CD163^+^ cells was much larger (Fig 4B, right column).

Immunohistochemical data were consistent with the results of western blot analysis. In the uninfected animals, CD68 was hardly detectable (Fig 5A and 5F). The increased CD68 protein levels were observed in all *O*. *felineus*–infected groups at all time p.i. The level of CD163 protein in the liver significantly increased (fourfold) compared with the uninfected hamsters only at 6 months p.i. (Fig 5E and 5F, P = 0.0009) and remained high at 1.5 years p.i. (p = 0.009). According to the linear regression model, a time-dependent increase in the level of CD163 protein was shown (R = 0.58; p = 0.022).

The TNF-α protein level was also at an undetectable level in the liver of uninfected animals (Fig 5B and 5F). Starting from 3 months p.i., the amount of the TNF-α protein was significantly higher than that in control animals (p = 0.007). The maximum level of this protein was found in animals at 1.5 years p.i. (p = 0.0005). According to the linear regression model, a time-dependent increase in the amount of TNF-α during the infection was demonstrated (R = 0.79; p < 0.001). It should be also noted, that a drop of TNF-α level at 6 months p.i. was insignificant (p = 0.62, Tukey test).

We also found that in the liver of infected hamsters at 1 month p.i., the level of the CK7 protein increased 5-fold (Fig 5D and 5F, p = 0.005). This protein is produced in epithelial cells, and its increased production in the liver of hamsters with opisthorchiasis supports the data on enhancement of epithelial hyperplasia. The level of CK7 as a marker of epithelial proliferation remained high throughout the time of the experimental infection.

Moreover, to assess fibrogenic processes, we quantified the α-SMA protein in liver tissues. An elevated level of α-SMA was found in the infected animals. The amount of this protein increased 7-fold already at 1 month p.i. (Fig 5C and 5F, p = 0.04). The level of α-SMA remained elevated throughout the experimental infection.

## Discussion

In this study, for the first time, we demonstrated that there is time-dependent accumulation of oxidative lesions in the hamster liver during *O*. *felineus* infection. In particular, we noted that as the infection progresses, the content of LPO byproducts HNE and MDA gradually increases. These changes in LPO markers correlated with the dynamics of pathological changes, when the magnitude of inflammation, epithelial hyperplasia, and cholangio- and periductal fibrosis gradually increased from the moment of infection to 1.5 years p.i.

During chronic inflammation, inflammatory cells are recruited to injury sites, thereby increasing the release and accumulation of free radicals. Lipid peroxides can be synthesized inside the cells, resulting in the formation of LPO byproducts HNE and MDA. HNE is the LPO byproduct most intensively studied in relation to its cytotoxic role promoting the development and progression of different pathological states [12]. The observed elevated ROS production during opisthorchiasis supports the recent reports that novel oxysterol derivatives have been identified in soluble extracts of *O*. *felineus* adult worms [8]. These oxysterol-like metabolites may possess mutagenic, genotoxic, pro-oxidative, and proinflammatory properties [8, 16, 17].

Endogenous free radicals generated by inflammatory and epithelial cells and from LPO products can induce the formation of oxidative and nitrative DNA lesion products [13]. Besides, we demonstrated that throughout the experimental *O*. *felineus* infection, the urinary level of 8-OHdG is elevated, which is one of the predominant forms of free-radical–induced highly mutagenic oxidative DNA lesions [18, 19].

Moreover, this study indicates that macrophages with different immunophenotypes participate in pathological changes in *O*. *felineus*–infected hamster livers. We detected elevated levels of CD68, COX2, TNF-α, and CD163 in *O*. *felineus*–infected golden hamsters. At the same time, we proved that the accumulation of proteins TNF-α and CD163 was time dependent.

There are data revealing the relation between macrophages of various immunophenotypes and myofibroblast activation during inflammation [20]. M1 macrophages generate cytokines that activate myofibroblasts possibly by the production of proinflammatory cytokines such as TNF-α. Alternatively, activated (CD163^+^) macrophages contribute to the control of inflammatory process through the release of anti-inflammatory cytokines: a process that promotes tissue regeneration [14, 15, 20]. On the other hand, during chronic *O*. *felineus* infection, chronic activation of (CD163^+^) macrophages presumably leads to the continuous production TGF-β and growth factors that promote proliferation of myofibroblasts, activation of epithelial–mesenchymal transition, extracellular matrix deposition, and fibrosis development. Myofibroblasts are characterized by α-SMA expression and an ability to deposit excessive amounts of extracellular-matrix components. To assess myofibroblast activation and fibrogenic processes, we measured the level of the α-SMA protein in liver tissues. We showed that the amount of this protein was elevated during the whole time of experimental opisthorchiasis, which manifested itself as excessive fibrogenesis as determined by histopathological examination. Atypical hyperplasia and dysplasia of the epithelium of the bile duct as well as egg granulomas in the periductal tissues are potential precancerous lesions. In this study, we demonstrated that the level of CK7 as a marker of epithelial hyperplasia is significantly higher than that in uninfected animals and remains high throughout the *O*. *felineus* infection time. These results confirm liver hyperplasia in opisthorchiasis.

The data we obtained are similar to the results on experimental animals and patients with other liver fluke infections [17, 21]. HNE and MDA were previously detected in mice infected by *C*. *sinensis* for 3 months [21] and in hepatitis C virus–associated liver diseases and hepatocellular carcinoma tissues, suggesting that oxidative stress may be related to various liver diseases [22]. Marked deregulation of an immune response and increased production of both proinflammatory and anti-inflammatory cytokines were detected previously in *O*. *viverrini*–associated CCA [23]. TNF-α participates in systemic inflammation, and it was reported that increased levels of TNF-α are associated with the hepatobiliary abnormalities in *O*. *viverrini*–infected hamsters [24]. CD163-expressing macrophages have been detected at sites of inflammation, such as chronically inflamed arthritis-affected joints and in the vicinity of tumor cells (tumor-associated macrophages) [15, 25], in particular in CCA associated with *O*. *viverrini* infection [25].

Thus, the pathogenesis of opisthorchiasis caused by *O*. *felineus* is in many ways similar to the pathogenesis of diseases caused by other carcinogenic liver flukes: *O*. *viverrini* and *C*. *sinensis* [2]. First, CCA development in model animals with *O*. *felineus* infection is similar to that of *O*. *viverrini* and *C*. *sinensis* infections [7]. In addition, liver damage in opisthorchiasis is similar to disorders caused by other liver flukes [2, 4]. Liver fluke excretory–secretory products might contribute to the progression of CCA [26]. Specifically, *O*. *viverrini* granulin stimulates proliferation of cholangiocytes and angiogenesis [26, 27], while thioredoxin peroxidase inhibits apoptosis [26]. Some *O*. *felineus* products are highly immunogenic [11], they can interact with the epithelium and accumulate inside the cells [10]. It should be also noted that liver fluke-induced CCA is a multifactorial etiology and other factors, such as Helicobacter infection within the biliary tract or presence of carcinogens and their precursors in food might enhance CCA development [26].

In conclusion, our present data provide first insights into the histopathological mechanism by which *O*. *felineus* infection causes liver injuries. *O*. *felineus*–associated chronic inflammation increases oxidative stress, which can overwhelm antioxidant system homeostasis to dampen ROS production and consequent oxidative modification of lipids, nucleic acids, and proteins. The phenotypical damage is closely related to biochemical aberrations including the accumulation of LPO products, activation of COX-2, and an increase in oxidative DNA damage. We showed here that opisthorchiasis felinea is characterized by time-dependent accumulation of LPO byproducts. There is also a direct time-dependent increase in the accumulation of macrophages with various immunophenotypes (related to TNF-α and CD163), which facilitates myofibroblast activation and cholangio- and periductal fibrogenesis. Thus, our data suggest that *O*. *felineus* has a carcinogenic potential and may be included in Group 1 of biological carcinogens, along with other carcinogenic liver flukes: *O*. *viverrini* and *C*. *sinensis*. All these hypotheses require further investigation and experimental validation.

## Supporting information

S1 Appendix Expression of CD68 and COX2 demonstrated by immunohistochemistry. (PDF)

S2 Appendix Expression of CD163 demonstrated by immunohistochemical analysis. (PDF)

S3 Appendix Images of western blot. (PDF)

S4 Appendix Urinary 8-hydroxy-2¢-deoxyguanosine (8-OHdG) levels in hamsters with opisthorchiasis. (XLSX)

S5 Appendix The values used to build Fig 3. (XLSX)

S6 Appendix The points extracted from western blot images. (XLSX)

S7 Appendix Densitometry normalized to b-actin (calculated from densitometry of WB images). (XLSX)

S8 Appendix The values used to build Fig 5. (XLSX)
