# Supplementary material for: Opisthorchis felineus infection provokes time-dependent accumulation of oxidative hepatobiliary lesions in the injured hamster liver
Source: PLoS One. 2019 May 14;14(5):e0216757. doi: 10.1371/journal.pone.0216757 (PMC6516637; doi:10.1371/journal.pone.0216757)
Supplement: S1 Appendix — (PDF) [file pone.0216757.s001.pdf]

# S1 Appendix

Intact

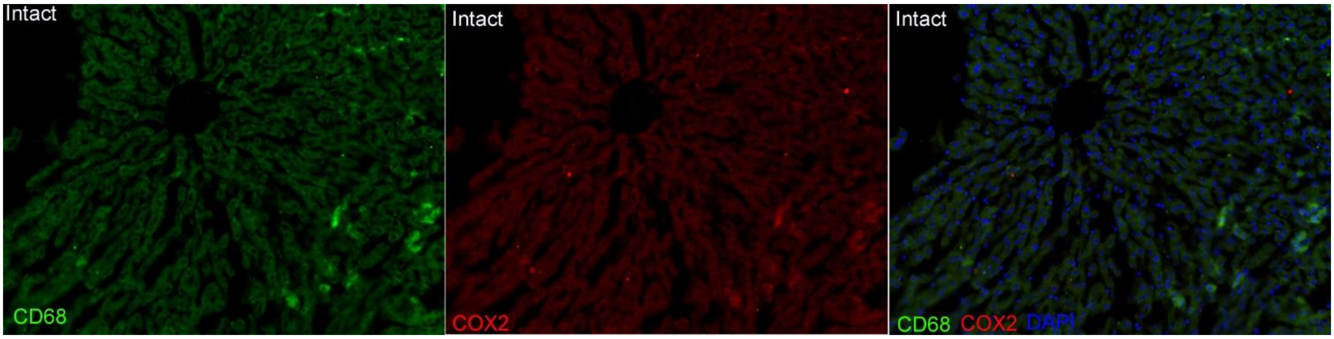


Opisthorchiasis felinea 1 month p.i.

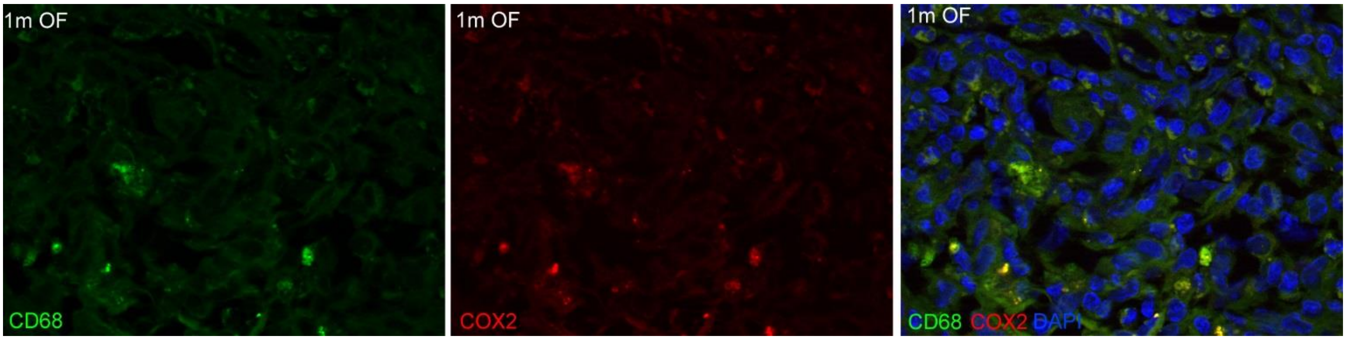


Opisthorchiasis felinea 1.5 year p.i.

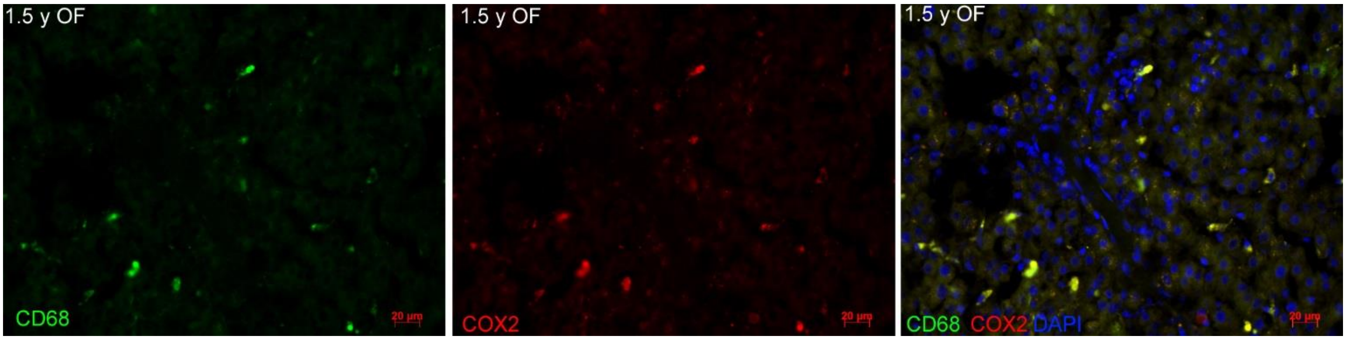


**Expression of CD68 and COX2 demonstrated by immunohistochemistry.** Pictures of lower magnifications are presented.
