# Supplementary material for: Opisthorchis felineus infection provokes time-dependent accumulation of oxidative hepatobiliary lesions in the injured hamster liver
Source: PLoS One. 2019 May 14;14(5):e0216757. doi: 10.1371/journal.pone.0216757 (PMC6516637; doi:10.1371/journal.pone.0216757)
Supplement: S2 Appendix — (PDF) [file pone.0216757.s002.pdf]

S2 Appendix

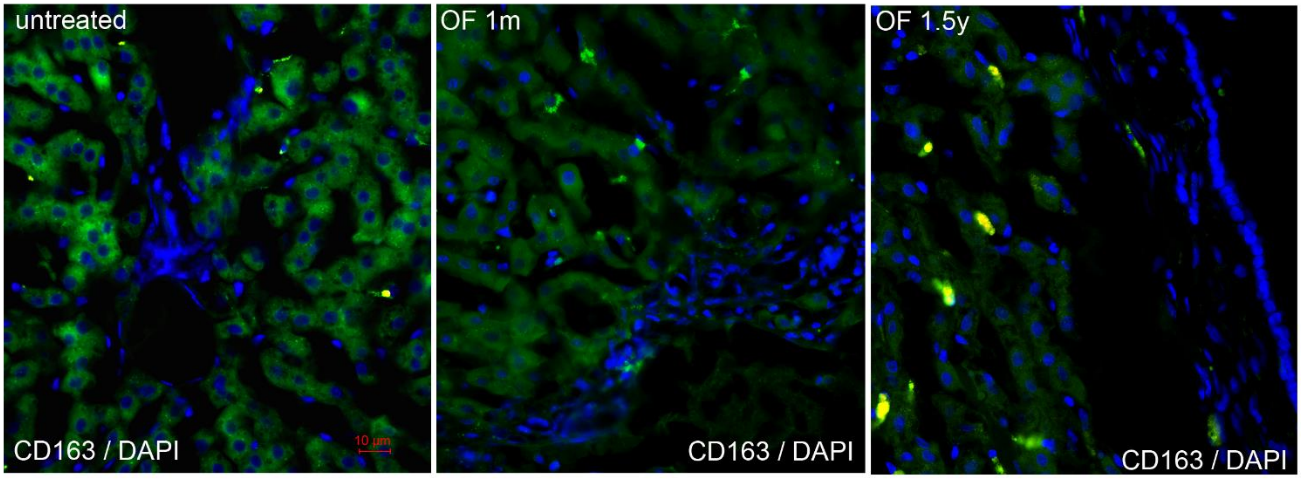

Expression of CD163 demonstrated by immunohistochemical analysis. Pictures of lower magnifications are presented.
