# Supplementary material for: Opisthorchis felineus infection provokes time-dependent accumulation of oxidative hepatobiliary lesions in the injured hamster liver
Source: PLoS One. 2019 May 14;14(5):e0216757. doi: 10.1371/journal.pone.0216757 (PMC6516637; doi:10.1371/journal.pone.0216757)
Supplement: S3 Appendix — (PDF) [file pone.0216757.s003.pdf]

S3 Appendix. Images of Western blot

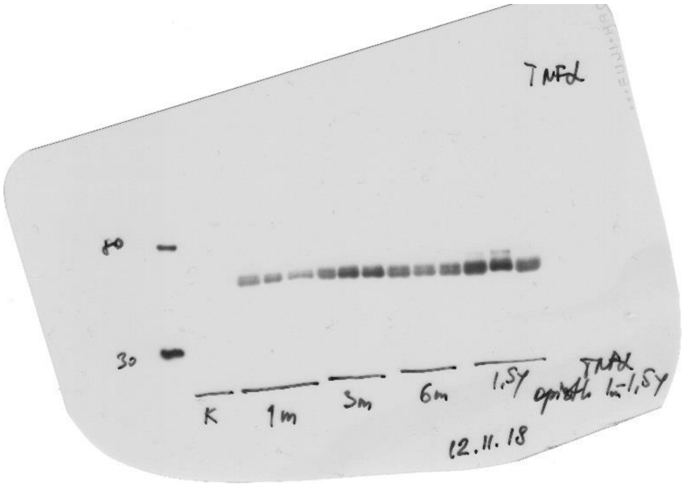

Tumor necrosis factor alfa,
TNFa

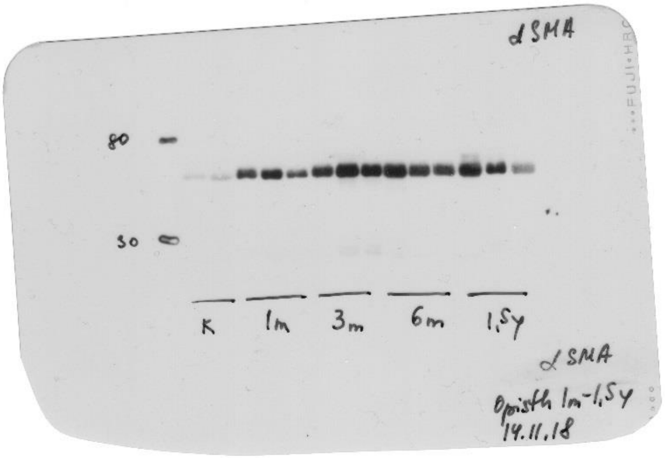

Alfa smooth muscle actin, aSMA

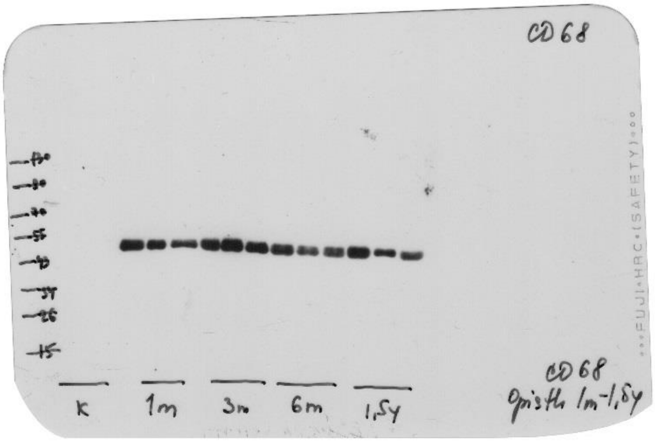

Cluster of Differentiation 68,
CD68

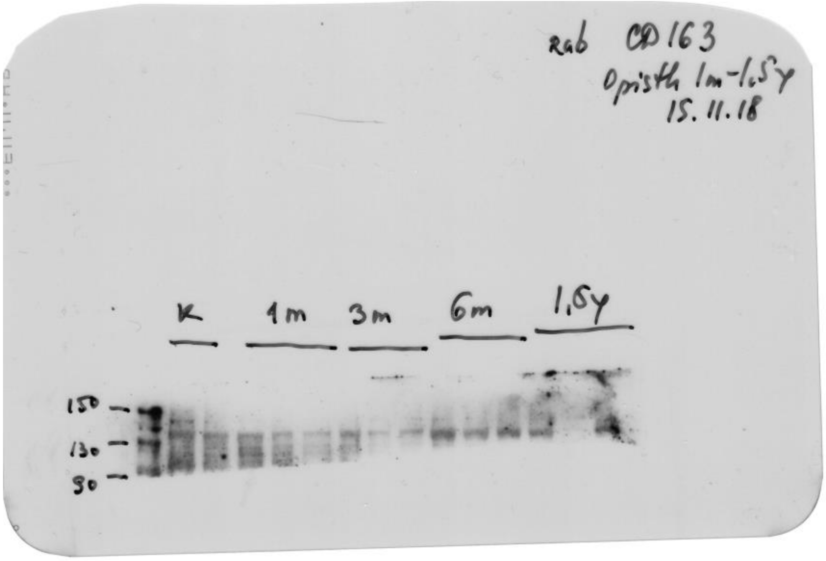

Cluster of Differentiation 163, CD163

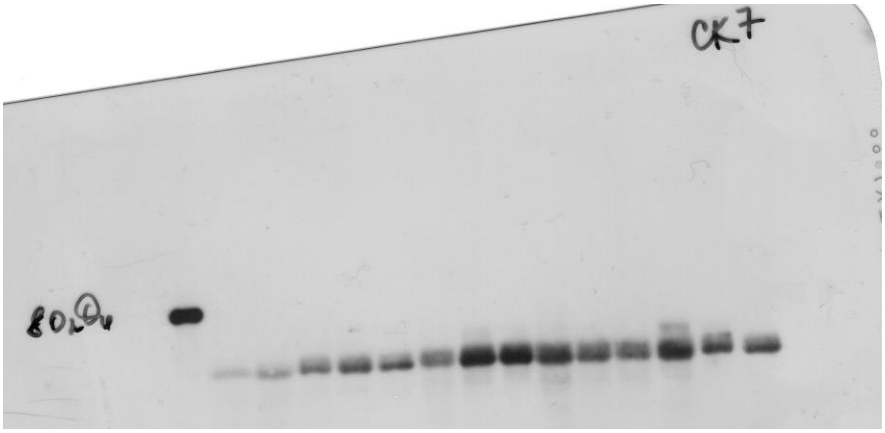

Cytokeratin 7, CK7

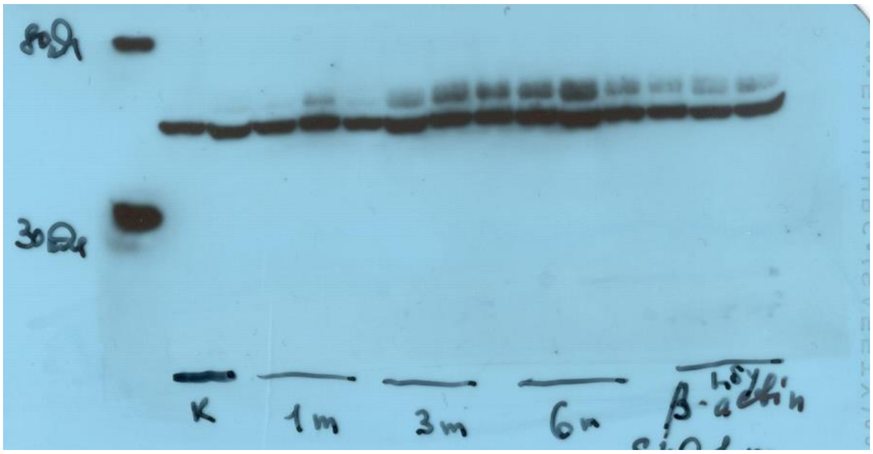

Beta-actin, b-actin
